# Molecular Changes in Prader-Willi Syndrome Neurons Reveals Clues About Increased Autism Susceptibility

**DOI:** 10.3389/fnmol.2021.747855

**Published:** 2021-10-29

**Authors:** A. Kaitlyn Victor, Martin Donaldson, Daniel Johnson, Winston Miller, Lawrence T. Reiter

**Affiliations:** ^1^IPBS Program, Neuroscience Institute, University of Tennessee Health Science Center, Memphis, TN, United States; ^2^Department of Neurology, University of Tennessee Health Science Center, Memphis, TN, United States; ^3^Department of Pediatric Dentistry and Community Oral Health, University of Tennessee Health Science Center, Memphis, TN, United States; ^4^Molecular Bioinformatics Core, University of Tennessee Health Science Center, Memphis, TN, United States; ^5^Department of Pediatrics, University of Tennessee Health Science Center, Memphis, TN, United States; ^6^Department of Anatomy and Neurobiology, University of Tennessee Health Science Center, Memphis, TN, United States

**Keywords:** autism (ASD), mitochondria, Prader-Willi syndrome (PWS), neurodevelopment, dental pulp stem cell (DPSC), mRNA seq, genomic disorders, neurogenetic syndrome

## Abstract

**Background:** Prader-Willi syndrome (PWS) is a neurodevelopmental disorder characterized by hormonal dysregulation, obesity, intellectual disability, and behavioral problems. Most PWS cases are caused by paternal interstitial deletions of 15q11.2-q13.1, while a smaller number of cases are caused by chromosome 15 maternal uniparental disomy (PW-UPD). Children with PW-UPD are at higher risk for developing autism spectrum disorder (ASD) than the neurotypical population. In this study, we used expression analysis of PW-UPD neurons to try to identify the molecular cause for increased autism risk.

**Methods:** Dental pulp stem cells (DPSC) from neurotypical control and PWS subjects were differentiated to neurons for mRNA sequencing. Significantly differentially expressed transcripts among all groups were identified. Downstream protein analysis including immunocytochemistry and immunoblots were performed to confirm the transcript level data and pathway enrichment findings.

**Results:** We identified 9 transcripts outside of the PWS critical region (15q11.2-q13.1) that may contribute to core PWS phenotypes. Moreover, we discovered a global reduction in mitochondrial transcripts in the PW-UPD + ASD group. We also found decreased mitochondrial abundance along with mitochondrial aggregates in the cell body and neural projections of +ASD neurons.

**Conclusion:** The 9 transcripts we identified common to all PWS subtypes may reveal PWS specific defects during neurodevelopment. Importantly, we found a global reduction in mitochondrial transcripts in PW-UPD + ASD neurons versus control and other PWS subtypes. We then confirmed mitochondrial defects in neurons from individuals with PWS at the cellular level. Quantification of this phenotype supports our hypothesis that the increased incidence of ASD in PW-UPD subjects may arise from mitochondrial defects in developing neurons.

## Introduction

Prader-Willi syndrome (PWS) is a multifaceted neurodevelopmental disorder characterized by hypotonia, hyperphagia, and developmental delay (Cassidy et al., 2012). Prader-Willi syndrome is caused by a loss of expression for one or more paternally expressed genes in the 15q11.2-q13.1 region (the PWS/AS critical region). Most PWS cases are caused by a paternal interstitial deletion in this region, but a smaller percentage of PWS is caused by the inheritance of two copies of maternal chromosome 15, maternal uniparental disomy (UPD) (Cheon, 2016). Due to imprinted expression of critical genes in the 15q11.2-q13.1 region, a set of normally paternally expressed genes are effectively silenced in neurons including *MAGEL2*, *SNORD115/116*, *SNRPN*, and *SNURF* (Angulo et al., 2015). Prader-Willi syndrome caused by UPD (PW-UPD) results in a milder phenotype than PWS caused by a paternal deletion (PW-del). However, UPD cases have a higher risk for autism spectrum disorder (ASD) than typically developing individuals (Whittington and Holland, 2010; Bennett et al., 2015; Dykens et al., 2017; Baker et al., 2018; Dimitropoulos et al., 2019) and later in life can develop cycloid psychosis (Verhoeven et al., 2003; Singh et al., 2019).

The goal of this study is to identify molecular changes that may confer increased autism incidence in PW-UPD subjects through the analysis of expression differences among neurons from PW-UPD, PW-del and control individuals. We used our large collection of dental pulp stem cell (DPSC) lines to generate neurons in culture for these studies (Goorha and Reiter, 2017; Victor and Reiter, 2017). DPSC are neural crest stem cells that reside inside the pulp cavity of naturally shed “baby teeth” (Gronthos et al., 2000, 2002; Miura et al., 2003). They are multipotent stem cells that have been differentiated to various cell types including neurons, osteocytes, glial cells, and adipocytes (Ikbale et al., 2016; Nuti et al., 2016; Young et al., 2016). In fact, several groups have shown that DPSC-derived neuronal cultures exhibit electrophysiological properties of functional neurons (Kiraly et al., 2009; Gervois et al., 2015; Urraca et al., 2015; Li et al., 2019). Previously, we established DPSC growth parameters (Urraca et al., 2015), efficacy (Wilson et al., 2015), and similarity to other stem cell systems (Victor and Reiter, 2017). In an earlier gene expression study of DPSC neurons from duplication 15q11.2-q13.1 (Dup15q) and Angelman syndrome (AS) deletion subjects, we identified distinct expression patterns indicative of each syndrome (Urraca et al., 2018). For the molecular studies presented here, we differentiated these stem cells into neuronal cultures using our previously published protocol (Goorha and Reiter, 2017). After differentiation, RNA sequencing (RNAseq) was performed to define transcriptional differences among PWS subtypes and neurotypical controls. These molecular studies revealed new details about shared gene expression changes in PWS and unique expression defects related to mitochondria in PWS-UPD neurons that may contribute to increased autism risk.

## Materials and Methods

### Obtaining Teeth for Dental Pulp Stem Cells Cultures

Neurotypical control teeth were obtained through the Department of Pediatric Dentistry and Community Oral Health at the University of Tennessee Health Science Center (UTHSC). Teeth from children with PWS subtypes were collected remotely by the caregivers of these subjects after confirmation of the underlying genetic diagnosis. Subjects provided informed consent for tooth collection along with a Social Communication Questionnaire (SCQ) to assess ASD status. Tooth pulp was cultured from teeth and cell lines frozen during early passages in our DPSC Repository as previously described (Goorha and Reiter, 2017). The DPSC Repository and subsequent molecular studies were approved by the UTHSC institutional review board prior to conducting research.

### Generation of Dental Pulp Stem Cell Cultures

Dental pulp stem cells (DPSC) used in this study were isolated and cultured according to our previously described protocol and stored in the DPSC Repository (Goorha and Reiter, 2017). Briefly, after mincing the dental pulp from inside the tooth cavity, 3 mg/mL Dispase II and 4 mg/mL Collagenase I were added to digest the tissue. Cells were then seeded on poly-D-Lysine coated 12-well plates with DMEM/F12 1:1, 10% fetal bovine serum (FBS), 10% newborn calf serum (NCS), and 100 U/mL penicillin and 100 ug/mL streptomycin (Pen/Strep) (Fisher Scientific, Waltham, MA). Confluent cultures (80%) were passaged with TrypLE^TM^ Express and neuronal differentiation performed only on early passage cells (<passage 4).

### Neuronal Differentiation

Dental pulp stem cells (DPSC) lines were seeded at 20,000 cells/cm^2^ on poly-D-lysine coated plates or chamber slides (Ibidi, Planegg, Germany) with DMEM/F12 1:1, 10% fetal bovine serum (FBS), 10% newborn calf serum (NCS), with 100 U/mL penicillin and 100 ug/mL streptomycin (Pen/Strep). At 80% confluence, the neuronal differentiation protocol was followed as previously published in Kiraly et al. (2009) with an extended maturation phase (3 weeks versus 7 days) (Goorha and Reiter, 2017). Briefly, epigenetic reprogramming was performed by exposing the DPSC to 10 μM 5-azacytidine (Acros Scientific, Geel, Belgium) in DMEM/F12 containing 2.5% FBS and 10 ng/mL bFGF (Fisher Scientific, Waltham, MA) for 48 h. Neural differentiation was induced by exposing the cells to 250 μM IBMX, 50 μM forskolin, 200 nM TPA, 1mM db-cAMP (Santa Cruz, Dallas, TX), 10 ng/mL bFGF (Invitrogen, Carlsbad, CA), 10 ng/mL NGF (Invitrogen, Carlsbad, CA), 30 ng/mL NT-3 (Peprotech, Rocky Hill, NJ), and 1% insulin-transferrin-sodium selenite premix (ITS) (Fisher Scientific, Waltham, MA) in DMEM/F12 for 3 days. At the end of the neural induction period, neuronal maturation was performed by maintaining the cells in Neurobasal A media (Fisher Scientific, Waltham, MA) with 1mM db-cAMP, 2% B27, 1% N2 supplement, 30 ng/mL NT-3, and 1X Glutamax (Fisher Scientific, Waltham, MA) for 21 days.

### RNA Sequencing of Dental Pulp Stem Cells-Neurons

Once DPSC-neurons were matured for 3 weeks, total RNA was collected using the Zymo Directzol RNA extraction kit (Zymo, Irvine, CA). Extracted RNA was assayed for quality and integrity using the Agilent Bioanalyzer 6000 pico chip (Agilent, Santa Clara, CA). Only RNA with an RNA Integrity Number (RIN) ≥ 9.0 was used for RNAseq studies. Library preparation and RNAseq was performed by Novogene (NovoSeq 6000) (Sacramento, CA) using the Illumina platform and paired end reads. 20M reads per sample were collected.

### RNAseq Analysis

FASTQ files from Novogene were analyzed for quality and trimmed using FASTQC. All reads were trimmed to remove nucleotides with Phred scores < Q20. The trimmed FASTQ files were aligned to the human genome reference library hg19 using RNASTAR (Widmann et al., 2012). Once aligned, the SAM files were collected and mined for read count information of each gene present in the reference file. Read counts were normalized using Counts per Million (CPM) method across groups for the entire experiment. Principle component analysis and Pearson’s coefficient plots were performed on the normalized transcriptome profile. A Wilcoxon’s *t*-test was used to determine significance across groups. All genes that fail to yield a p-value ≤ 0.05 and a fold change greater than 1.5 were removed. Benjamini and Hochberg false discovery rate (FDR) was performed on this trimmed gene list. All genes that failed to yield an FDR rate of ≤ 0.05 were removed. The final significant differential gene lists were loaded into the ClustVis web tool to generate heatmaps (Metsalu and Vilo, 2015). Additionally, the targets were loaded into the web based enrichment analysis tool, Database for Annotation, Visualization and Integrated Discovery (DAVID) (Huang da et al., 2009b) to identify enriched gene ontology (GO) terms.

### Immunofluorescence

Dental pulp stem cell (DPSC) were grown and differentiated on 3-well chamber slides (Ibidi, Planegg, Germany) coated with poly-D-lysine. Cells were fixed using a 4% paraformaldehyde solution for 10 min. Once fixed, cells were blocked and permeabilized using PBS with 1% BSA, 10% FBS, and 0.3% Triton X-100 for 1 h. Primary antibodies were diluted to 1:500 for anti-Beta Tubulin (Millipore, ab9354) and anti-TOMM20 (Santa Cruz, sc-17764) in the blocking solution and incubated overnight at 2–8°C with agitation. After overnight incubation, the slides were washed 3x with PBS-T for 10 min before the secondary antibodies, Goat anti-mouse Alexa Fluor 488 (Life Technologies, A11029) and Goat anti-chicken Alexa Fluor 594 (Life Technologies, A11042) were added at a 1:1000 dilution. Slides were incubated at room temperature for 1 h and then washed again 3× for 10 min each. Finally, Prolong Gold Antifade with DAPI (Fisher Scientific, Waltham, MA) was applied for mounting. Slides were imaged on a Zeiss 710 confocal microscope at 63× magnification using Z-stacking to image the entire neuron via ZEN software (Black Edition).

### Western Blots

Western blots were performed as previously described (Urraca et al., 2018). Briefly, protein was extracted from 3-week mature neurons using neuronal protein extraction reagent (N-PER) (Fisher Scientific, Waltham, MA) and protease inhibitor cocktail (Roche). Samples were resolved on a NuPage 1.5mm 4–12% Bis-Tris gel (Invitrogen, Carlsbad, CA) according to manufacturer instructions and transferred to an Invitrolon^TM^ -PVDF membrane (Invitrogen, Carlsbad, CA). The membrane was blocked using Odyssey Blocking Buffer (Licor, Lincoln, NE) for 1 h and incubated overnight at 2–8°C in primary antibody with agitation. Primary antibodies used: anti-MAP2 (Santa Cruz, sc-32791), anti-Nestin (Santa Cruz, sc-23927), and anti-GABA A receptor beta 3 (Abcam, ab98968). anti-GAPDH (Abcam, ab157156) was used as a protein loading control. Blots were incubated at room temperature for 1 h in secondary antibodies for both the 700 and 800 channels using Li-Cor IR secondary antibodies (Licor, 926-32212 and 926-68074). Blots were imaged on a Li-Cor Odyssey^TM^ Fc Imager. Both the 700 and 800 channels were exposed for 2 min.

### Image Analysis

All imaging analysis was done using coded cell lines, so the observer was blinded to the genotypes. Only after data collection and analysis were cell lines decoded. For each cell line (≥ 5 individuals per group), ≥15 neurons were imaged for analysis of mitochondrial abundance within the neuron. To quantify mitochondrial area, cells were labeled with anti-Beta Tubulin (total neuronal area) and anti-TOMM20 (mitochondrial area), images were loaded into the Imaris imaging analysis software (Oxford Instruments, Abingdon, United Kingdom) and surfaces for each marker were created (see Figure 6). The mitochondrial area (anti-TOMM20) was then divided by the total neuronal area (anti-Beta Tubulin) to give the percentage of the neuron that contains mitochondria. After collecting all data, significance testing was performed by both ordinary one-way ANOVA and Turkey’s multiple comparisons test with a single pooled variance.

**FIGURE 1:**
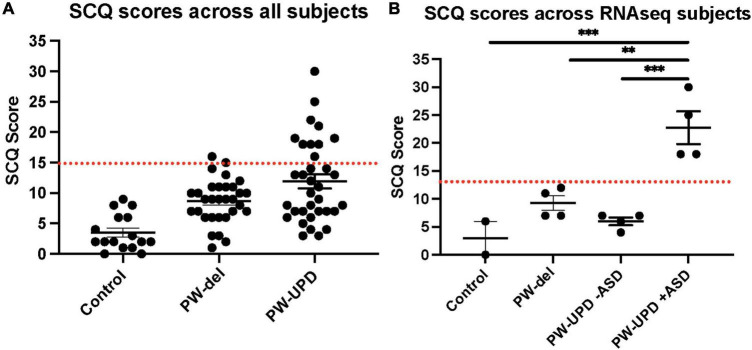
Social Communication Questionnaire (SCQ) scores across all subjects. **(A)** SCQ scores across all subjects. **(B)** SCQ scores across individuals used for RNAseq analysis. A cutoff score of 15 (red line) is used as a threshold for “possible ASD.” The PW-UPD cohort has two segregated groups. Subjects that meet the criteria for “Possible ASD” (score of 15 or above) and those scoring within normal limits. For 2 of the neurotypical control subjects used, SCQs could not be collected. Significance testing was performed by one-way ANOVA testing. ** = *p*-value ≤ 0.01, *** = *p*-value ≤ 0.005.

**FIGURE 2:**
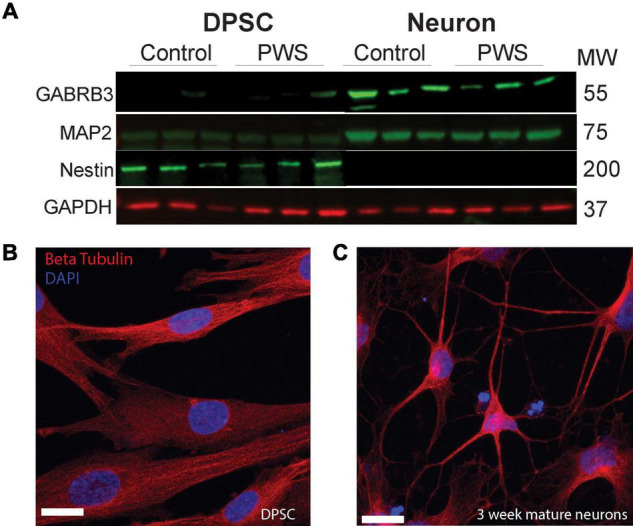
DPSC from both PWS and control subjects differentiate efficiently into neuronal cultures. **(A)** DPSC from all subjects are negative for neuronal markers, MAP2 and GABA A, but positive for stem cell marker NESTIN. After differentiation and 3 weeks of maturation, the cultures are positive for both MAP2 and GABA A receptor subunit beta 3. **(B,C)** Representative DPSC versus DPSC-derived neurons visualized with anti-beta tubulin (red) **(B)** DPSC show a flat fibroblast-like morphology as previously reported. **(C)** DPSC-derived neuronal culture contains cells with pyramidal neuron morphology. DAPI (blue) was used as a nuclear stain. 63× magnification. Scale bar is 20 μM.

**FIGURE 3:**
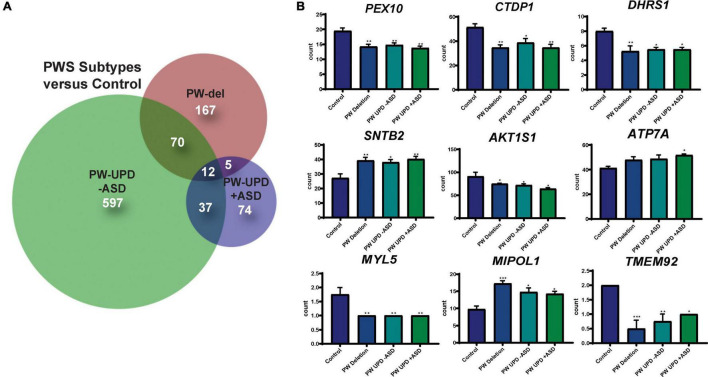
DPSC-derived neuronal cultures define PWS subtype specific expression and a core PWS molecular signature. **(A)** Venn diagram using all the significantly differentially expressed transcripts (p-value ≤ 0.05, FDR ≤ 0.05) versus control for each PWS subtype created using BioVenn (Hulsen et al., 2008). **(B)** Individual bar graphs showing mean counts per transcript across all groups for each of the transcripts located outside of the PWS critical region and identified as common across all PWS subtypes. These 9 genes, along with *MAGEL2, SNRPN*, and *SNURF*, represent a core molecular signature for PWS associated changes in neurons. Significance was determined by individual *t*-tests versus control for each subgroup (*p* ≤ 0.05). * = *p*-value ≤ 0.05, ** = *p*-value ≤ 0.01, *** = *p*-value ≤ 0.005.

**FIGURE 4:**
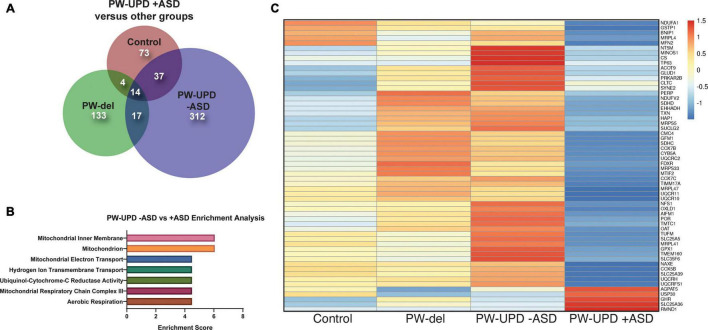
RNAseq analysis reveals enrichment in differentially expressed mitochondrial transcripts in the PW-UPD + ASD group. **(A)** Venn diagram using all the significantly differentially expressed transcripts (p-value ≤ 0.05, FDR ≤ 0.05) versus PW-UPD + ASD for each PWS subtype and control created using BioVenn (Hulsen et al., 2008). **(B)** The list of differentially expressed transcripts PW-UPD -ASD versus UPD + ASD (p-value ≤ 0.05, FDR ≤ 0.05) was used as input for DAVID (Huang da et al., 2009a, b) enrichment analysis. The top enrichments (enrichment score ≥ 3.0) were transcripts located within mitochondria and having mitochondrial functions (p-value ≤ 0.002). **(C)** Heatmap of transcripts identified by DAVID analysis as mitochondrial related. Expression of genes identified as driving the mitochondria enrichment in the PW-UPD + ASD group were used to create this heatmap across all subgroups (Metsalu and Vilo, 2015). Colors indicate read counts from high (red) to low (blue). Most of the mitochondrial transcripts identified in DAVID enrichment set have reduced expression in the PW-UPD + ASD group compared to all other groups.

**FIGURE 5:**
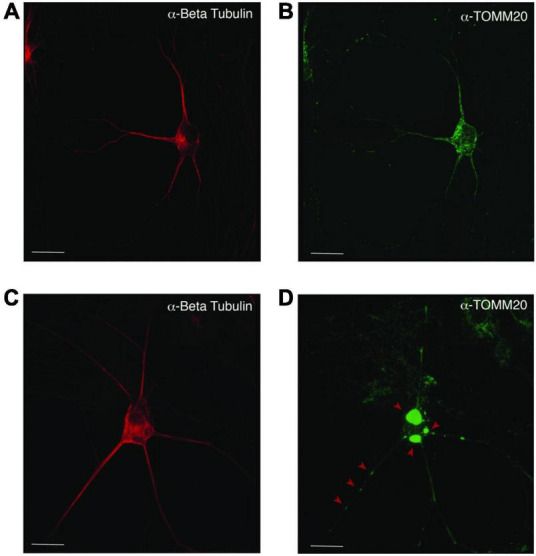
Mislocalization and reduced dispersion of mitochondria in ASD neurons. Neuronal cultures visualized with anti-beta tubulin (red) and the mitochondrial marker anti-TOMM20 (green). The PW-UPD -ASD neuron (top row) shows bright and evenly dispersed mitochondria within the neuronal projections. In PW-UPD + ASD neuron, the red arrows point to mitochondrial aggregates not seen in the PW-UPD -ASD neurons. **(A,C)** show neuronal morphology using anti-beta tubulin staining. **(B,D)** show the anti-TOMM20 staining to identify mitochondria. Confocal stacks were taken at 63X magnification. Scale bar is 20 μm.

**FIGURE 6:**
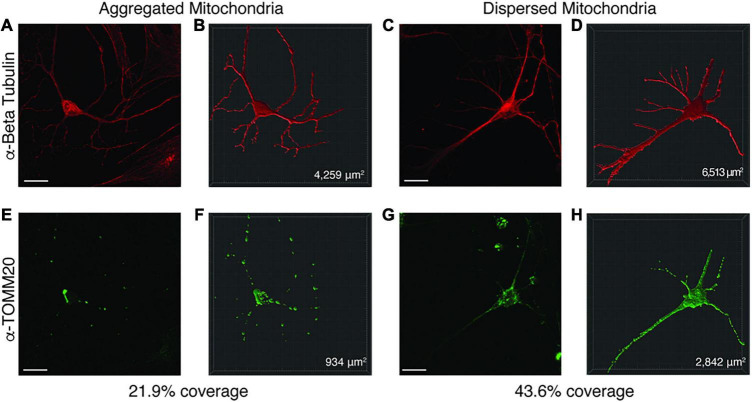
Representative image of Imaris volumetric analysis. Z stacks from neuronal images taken on a Zeiss 810 confocal microscope were loaded into the Imaris software suite for analysis. **(A,E)** show a representative image of a neuron with mitochondrial aggregates while **(C,G)** depict a neuron with dispersed mitochondria. Anti-Beta Tubulin **(A,C)** was used to measure the surface area of the neuron, while anti-TOMM20 **(E,G)** was used to label total mitochondria content within the neuron. Using the surfaces function in Imaris, a mask for the Beta Tubulin **(B,D)** and TOMM20 **(F,H)** stained areas was constructed. From these surface masks, the area is calculated by the software (bottom right corner of **B,D,F**,**H**) for each surface. The mitochondrial area within the neuron was divided by the total neuronal area to determine a percentage of mitochondrial volume. Images were taken at 63× by confocal microscopy. Scale bar represents 20 μM.

## Results

### Prader-Willi Uniparental Disomy Subjects Have an Increased Incidence of Autism Spectrum Disorder Versus Paternal Deletion and Control Subjects

Several studies have shown an incidence of about 35% for ASD in PW-UPD subjects compared to 18% in PW-del subjects (Bennett et al., 2015; Baker et al., 2018; Dimitropoulos et al., 2019). To measure ASD remotely, we used the Social Communication Questionnaire (SCQ). The SCQ provides a way to ascertain the likelihood a subject may have ASD, especially when face to face evaluation is not possible. Several groups have now validated the SCQ as a screening tool for ASD against other tools like the Autism Diagnostic Interview, Revised (ADI-R) (Hutchins et al., 2007; Sappok et al., 2015; Barnard-Brak et al., 2016; Chesnut et al., 2017; Mouti et al., 2019). We use the “lifetime” version of the test which has been proven to be a more accurate predictor of ASD (Chesnut et al., 2017). A cutoff score of 15 is considered “possible ASD” according the SCQ lifetime tool. We selected 4 lines from each group (neurotypical control, PW-del, PW-UPD -ASD, and PW-UPD + ASD) for RNAseq analysis and additional lines were used for the immunofluorescence analysis. Figure 1 shows the SCQ scores from these subjects, except for two neurotypical control subjects for which we did not receive an SCQ. For one of the neurotypical control subjects without SCQ data, we did collect the Social Responsiveness Scale (SRS) which revealed the subject did not have any autism characteristics. The PW-UPD group shows two distinct clusters, those scoring above 15 (possible ASD) and those within normal limits (Figure 1).

### Characteristics of Mixed Neuronal Cultures Derived From Control and Prader-Willi Syndrome Dental Pulp Stem Cells Lines

Four DPSC lines representing each of the PWS subgroups (PW-del, PW-UPD -ASD, and PW-UPD + ASD) and 4 neurotypical control subject lines were selected from the repository for RNAseq studies (Supplementary Table 1). Deciduous (“baby”) teeth from neurotypical control subjects were collected locally from the Department of Pediatric Dentistry and Community Oral Health at UTHSC and PWS subjects were collected remotely by parents and guardians of the subjects using our previously published protocols (Goorha and Reiter, 2017). DPSC lines were differentiated into neurons using a published neuronal differentiation protocol (Kiraly et al., 2009; Goorha and Reiter, 2017). DPSC from all groups show positive expression for the stem cell marker, NESTIN, while these same lines were negative for neuronal markers MAP2 and GABA A receptor subunit beta 3 (Figure 2A). After the neuronal differentiation protocol and a 3-week maturation period, the cultures were positive for both MAP2 and GABA A receptor subunit beta 3 (Figure 2A). Dental pulp stem cells show a flat, fibroblast like morphology (Figure 2B), but after a month of differentiation, the cultures display a pyramidal neuron-like morphology (Figure 2C). Both neurotypical and PWS neuronal cultures displayed similar morphologies and cell numbers throughout the differentiation process. We have previously established that a 3-week neuronal maturation period is sufficient to induce significant gene expression changes indicative of terminal neuronal differentiation (Urraca et al., 2015) and reflect underlying disease specific gene expression patterns (Urraca et al., 2018).

### Prader-Willi Syndrome Subtypes Show Distinct Transcriptional Profiles and a Shared Prader-Willi Syndrome Expression Signature That Extends to Genes Outside of the Prader-Willi Syndrome Critical Region (15q11.2-q13.1)

RNAseq files from each individual were analyzed by the UTHSC Bioinformatics Core to create lists of significantly (p-value ≤ 0.05 and FDR ≤ 0.05) different transcripts for each PWS subtype versus control and each subtype versus the other genotypes (see methods for details). Using these lists of significantly differentially expressed transcripts for each group versus control, we created Venn diagrams in BioVenn (Hulsen et al., 2008) (Figure 3A). From this analysis we identified a unique molecular signature for each subgroup and a core PWS signature comprised of 3 transcripts within the PWS critical region (*SNRPN*, *SNURF*, and *MAGEL2*) and 9 transcripts coding for genes located outside of 15q PWS/AS critical region (Figure 3B). Supplementary Table 2 lists these PWS specific transcripts, their function, and evidence that ties these genes or their function to PWS phenotypes. Analyzing the expression of genes across the PWS/AS critical region in our subjects shows the expected expression changes from control to PWS subtypes (Supplementary Figure 1). Specifically, expression of genes that are maternally imprinted in PWS (*MAGEL2*, *SNRPN*, *SNURF*, *NDN*, and *MRKN3*) are absent in our PWS groups, regardless of genetic subtype. These data confirm that DPSC neurons from subjects accurately recapitulate the genetic landscape of PWS in terms of imprinted gene expression.

### Significant Down Regulation of Mitochondrial Transcripts in Prader-Willi Syndrome Uniparental Disomy + Autism Spectrum Disorder Neurons

In order to find molecular changes specific to ASD in our dataset, we investigated the significantly different transcripts (p-value ≤ 0.05, FDR ≤ 0.05) in the -ASD groups versus the UPD + ASD group (Figure 4A). 380 transcripts met our significance cut off and were interrogated for enrichment analysis using the **D**atabase for **A**nnotation, **V**isualization and **I**ntegrated **D**iscovery (DAVID), which assigns enrichment scores for Gene Ontology (GO) and other descriptive terms significantly over-represented in our differentially expressed dataset as compared to the entire human genome (Huang da et al., 2009a, b). Using DAVID functional clustering for GO terms, we found significant enrichments in mitochondrial compartments, functions, and processes (p-value ≤ 0.002) (Figure 4B). The top enrichment clusters were mitochondrial membrane transcripts and transcripts involved in mitochondrial maintenance and biogenesis. It is important to note that no other enrichments (score ≥ 3.0) were found in this dataset. From the transcripts identified in the DAVID enrichment analysis, we created a heatmap across all groups for the mitochondrial genes responsible for the enrichment scores using ClustVis (Metsalu and Vilo, 2015) (Figure 4C). This heatmap illustrates that most mitochondrial transcripts identified in our enrichment analysis are dramatically decreased in the PW-UPD + ASD group. Supplementary Table 3 lists these transcripts and their function. These data support our premise that mitochondrial dysfunction may contribute to increased ASD risk in PW-UPD sub-group.

### Prader-Willi Uniparental Disomy + Autism Spectrum Disorder Neurons Display Cellular Level Defects in Mitochondria

**FIGURE 7:**
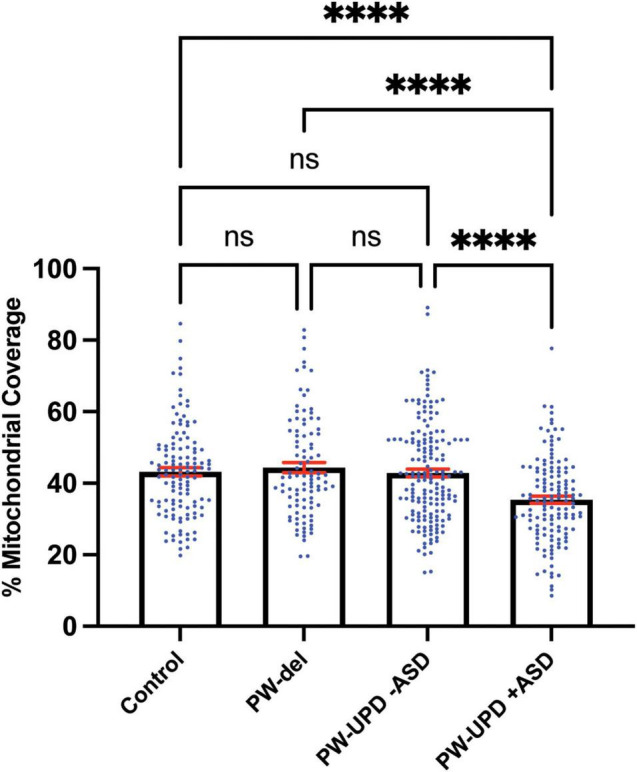
PW-UPD + ASD neurons show decreased mitochondrial coverage within neuronal area. Using Imaris, the total neuronal area (anti-Beta Tubulin) that contains mitochondria (anti-TOMM20) was calculated. Percent of mitochondrial coverage was measured for ≥15 neurons from each cell line and at least 5 cell lines per group in a blinded fashion. Significance testing was performed by both ordinary one-way ANOVA and Turkey’s multiple comparisons test with a single pooled variance. ns = not significant, **** = *p*-value ≤ 0.001.

Since enrichment analysis revealed global down regulation of mitochondrial transcripts, we looked for mitochondrial defects at the cellular level in PW-UPD + ASD neurons. Anti-Beta Tubulin was used to outline the area of the neurons (Figures 5A,C). To visualize mitochondria in neurons, we used the mitochondrial surface marker TOMM20 (translocase of outer mitochondrial membrane 20) (Lavie et al., 2017; Nguyen et al., 2018). Anti-TOMM20 revealed a mitochondrial phenotype within the + ASD neurons characterized by perinuclear aggregation of mitochondria and decreased mitochondria detected in the neuronal projections (Figures 5B vs. D). To quantify this mitochondrial specific phenotype, we performed a blinded analysis of neuron images from neurotypical control subjects and PWS subjects. For each group ≥5 individuals were used. Each cell line was coded to ensure no user bias when measuring the mitochondrial area within the neuron. The neuronal cultures were immunolabeled with anti-TOMM20 and anti-Beta Tubulin and then imaged through the cell by confocal microscopy. To determine the percentage of neuronal area containing mitochondria, Imaris software (Oxford Instruments, Abingdon, United Kingdom) was used on the confocal stacks. Neuron images were uploaded to the Imaris program and surfaces for quantification were created using the anti-Beta Tubulin (Figures 6A,C) signal to compute total neuronal area and anti-TOMM20 (Figures 6E,G) to compute the mitochondrial volume within the neuron. Figure 6 shows the Imaris output after using the “surfaces” function to define the area of both components. We were able visually to confine our measurements to single neurons and eliminate any debris from this analysis. For each cell line, ≥15 neurons were imaged and analyzed. We found a significant difference in the average neuronal mitochondrial coverage between the PW-UPD + ASD group and the other groups (Figure 7). These data confirm the hypothesis that mitochondrial dysfunction may underlie the increased ASD incidence of the UPD class.

## Discussion

Here we used our DPSC-derived neuronal cultures to define molecular signatures for each genomic subtype of PWS, providing for the first time a molecular signature of gene expression in PWS neurons as well as unique molecular changes in the PW-UPD + ASD group that may be indicative of an underlying mitochondrial defect related to autism. DPSC provide an excellent stem cell source to study neurodevelopmental disorders *in vitro* (Victor and Reiter, 2017). They are easy to collect remotely and provide a non-invasive way to obtain patient stem cells from a large number of individuals. In addition, DPSC have been found to more closely mimic the epigenetic landscape of embryonic stem cells compared other stem cell types commonly used for neurogenetic research, such as induced pluripotent stem cells (iPSC) (Dunaway et al., 2017).

Expression profiling in this study revealed transcripts that may affect early development in PWS, including genes involved in secretory granule regulation (*SNTB2* and *ATP7A*), mTOR signaling (*AKT1S1*), and peroxisome biogenesis (*PEX10*), all of which have been implicated in phenotypes of neurodevelopmental disorders (Niciu et al., 2006; Wang et al., 2017; Barone et al., 2019). Specifically, prohormone and neuropeptide secretion deficiencies are hallmarks of PWS (Burnett et al., 2017; Chen et al., 2020). These processes rely on efficient secretory granule production and regulation, both of which can be altered by *ATP7A* or *SNTB2*, through its interaction with *PTPRN* (Suckale and Solimena, 2010). We had previously shown that these MAGEL2 regulated secretory granule defects can be studied in DPSC derived PWS subject neurons. These secretory granule defects have been implicated as a cause of the hormone and neuropeptide deficiencies seen in PWS (Chen et al., 2020). *ATP7A* is a copper pump and is integral in regulating copper release at the synapse as well as modulating proteins in the secretory pathway (D’Ambrosi and Rossi, 2015). Due to its widespread function in the brain, it is not surprising that mutations in this gene lead to neurodegenerative and neurodevelopmental disorders (Kaler, 1993). Downstream investigation of these transcripts at the protein level in PWS neurons will be critical to understanding the effect abnormal expression levels of *ATP7A* or *SNTB2* may have on PWS neuropathological development and symptomology. Understanding the function of these genes in the context of PWS could lead to potential therapeutic targets for future interventions involving rescue of endocytic recycling defects driven by loss of MAGEL2 (Chen et al., 2020).

In addition to uncovering an expression signature common among PWS subtypes compared to neurotypical controls, we also identified transcriptional differences between PW-UPD -ASD and + ASD that may parallel the observed increased autism risk in the PW-UPD cases. In our own cohort of PWS subjects, 29% of PW-UPD individuals scored above the threshold for “possible ASD” on the SCQ (10 out of 34 subjects). This number is comparable to the 35% ASD incidence found in PW-UPD clinical cases (Bennett et al., 2015; Dimitropoulos et al., 2019). The SCQ has been validated against other ASD tests like ADI-R (Hutchins et al., 2007; Schanding et al., 2012; Sappok et al., 2015; Barnard-Brak et al., 2016; Chesnut et al., 2017) and provides at least an indicator of who may have ASD symptoms.

We identified 380 transcripts that were significantly differentially expressed between + ASD and -ASD groups for PW-UPD subjects (Figure 4A). Enrichment analysis on these 380 transcripts revealed overrepresented GO terms in mitochondrial compartments, functions, and pathways (Figure 4B). Many of the transcripts involved in these enrichments produce proteins that are part of the ETC complexes and proteins involved in mitochondrial function, maintenance and biogenesis (Supplementary Table 2) (Cogliati et al., 2018; Guo et al., 2018). Cellular analysis in neurons indicated that + ASD neurons showed a mitochondrial aggregation phenotype (Figure 5) and have significantly less mitochondrial volume within the neuron (Figure 7). This further supports our hypothesis that mitochondrial dysfunction (MD) may be involved in the increased ASD incidence in PW-UPD subjects. Additionally, MD has previously been identified in PWS fibroblasts and significant differences in mitochondrial respiration between PW-del and PW-UPD have previously been observed (Butler et al., 2018).

MD has been implicated in both idiopathic (Weissman et al., 2008) and syndromic forms of ASD, including Fragile X (Abbeduto et al., 2019), Down syndrome (Izzo et al., 2018), Rett syndrome (Hirofuji et al., 2018), and tuberous sclerosis (Ebrahimi-Fakhari et al., 2016). In fact, it has been reported that up to 80% of individuals with ASD may also have MD (Rose et al., 2018). Neurons consume a large amount of energy to maintain ionic gradients and support neurotransmission (Li et al., 2004; Kann and Kovacs, 2007; Princz et al., 2018) so it is not surprising that mitochondrial defects have been implicated in both neurodevelopmental and neurodegenerative disorders (Lin and Beal, 2006; Rossignol and Frye, 2012; Burté et al., 2015; Rose et al., 2018; Johnson et al., 2021; Rojas-Charry et al., 2021). Inside neurons, mitochondria are the main energy producers and are integral in maintaining calcium homeostasis at the synapse, which is vital for regulating neurotransmission (Lin and Sheng, 2015). Children diagnosed with both ASD and mitochondrial dysfunction (MD) have a higher rate of neurodevelopmental regression, seizures, and gross motor delay (Poling et al., 2006; Shoffner et al., 2010). Several studies have also shown abnormal levels of metabolic biomarkers such as pyruvate, lactate, glutathione, and ubiquinone in ASD subjects (Laszlo et al., 1994; Weissman et al., 2008; Essa et al., 2012; Khemakhem et al., 2017). Reduced activities in the electron transport chain (ETC), specifically complexes I and V, have been found in the frontal cortex of ASD individuals (Gu et al., 2013). Other studies using post-mortem ASD brain tissue found similar reductions across brain regions of mitochondrial respiratory transcripts and proteins (Anitha et al., 2012, 2013; Ginsberg et al., 2012). These studies in post-mortem idiopathic ASD brain, although not ideal, add to the evidence that MD may be one of the root causes of ASD in PW-UPD cases.

Performing rescue experiments to re-activate mitochondrial biogenesis, as we did for the restoration of endocytic recycling defects (Chen et al., 2020), will be a critical future study in our DPSC neuron system. These studies may lead to drug interventions already developed for mitochondrial disorders like Leigh syndrome. In particular, we found a significant decrease in the *PPARGC1A* gene (Supplementary Figure 2) in the PW-UPD + ASD subjects. The protein produced by this transcript, PGC1α, is a transcriptional coactivator that is responsible for regulating mitochondrial biogenesis (Ventura-Clapier et al., 2008; Jornayvaz and Shulman, 2010; Scarpulla, 2011). Using pharmacological agonists already shown to increase PGC1α expression (Zhang et al., 2013; Xu et al., 2018), drug screens can be performed to restore the mitochondrial phenotype we found in the PW-UPD + ASD neurons. Coupled with bioenergetic studies of living mitochondria in our PWS neuronal cultures, we will be able to determine the global effects on mitochondrial function caused by paternal loss of expression in PWS subjects.

## Conclusion

In this study we utilized our unique collection of patient-derived DPSC lines to generate neurons for next generation RNAseq analysis from three major genomic subtypes of PWS and compared them to neurotypical control neurons. We found 9 genes outside the PWS/AS critical region on 15q11.2-q13.1 that may contribute to the overall PWS phenotype. We also identified an expression signature in the PW-UPD + ASD group compared to all other groups that appears to indicate a global down regulation of mitochondrial genes is associated with ASD in the PW-UPD subjects. Finally, we showed that this molecular signature translated to a visible and quantifiable change in the appearance and abundance of mitochondria in PW-UPD + ASD neurons. These studies are the first steps at investigating the molecular defects in PWS neurons contributing to both PWS symptomology and increased autism incidence in PW-UPD cases.

## Data Availability Statement

The datasets presented in this study can be found in online repositories. The names of the repository/repositories and accession number(s) can be found below: https://www.ncbi.nlm.nih.gov/geo/, GSE178687.

## Ethics Statement

The studies involving human participants were reviewed and approved by the University of Tennessee Health Science Center Institutional Review Board. Written informed consent to participate in this study was provided by the participants’ legal guardian/next of kin.

## Author Contributions

LR conceived and acquired funding for this study. AV grew cultures and performed experiments. DJ and WM performed bioinformatic analysis. AV and LR analyzed data and wrote the manuscript. MD obtained neurotypical control teeth. All authors contributed to the article and approved the submitted version.

## Conflict of Interest

The authors declare that the research was conducted in the absence of any commercial or financial relationships that could be construed as a potential conflict of interest.

## Publisher’s Note

All claims expressed in this article are solely those of the authors and do not necessarily represent those of their affiliated organizations, or those of the publisher, the editors and the reviewers. Any product that may be evaluated in this article, or claim that may be made by its manufacturer, is not guaranteed or endorsed by the publisher.
