# Supplementary material for: Molecular Changes in Prader-Willi Syndrome Neurons Reveals Clues About Increased Autism Susceptibility
Source: Front Mol Neurosci. 2021 Oct 29;14:747855. doi: 10.3389/fnmol.2021.747855 (PMC8586424; doi:10.3389/fnmol.2021.747855)
Supplement: Supplementary file 1 [file Data_Sheet_1.pdf]

Supplementary Material

1 Supplementary Tables

Cell Line	Diagnosis	Sex	ASD Status
38*	N/A	F	Unknown
182*	N/A	F	Normal
189*	N/A	F	Unknown
238*	N/A	M	Normal
195	N/A	M	Normal
312	N/A	M	Normal
152*	PW deletion	M	Normal
192*	PW deletion	F	Normal
225*	PW deletion	M	Normal
258*	PW deletion	F	Normal
148	PW deletion	M	Normal
307	PW deletion	F	Normal
156*	PW UPD	F	Normal
162*	PW UPD	F	Normal
171*	PW UPD	F	Normal
249*	PW UPD	F	Normal
270	PW UPD	F	Normal

191	PW UPD	M	Normal
235	PW UPD	M	Normal
197	PW UPD	M	Normal
94*	PW UPD	F	Possible ASD
228*	PW UPD	M	Possible ASD
250*	PW UPD	M	Possible ASD
268*	PW UPD	F	Possible ASD
277	PW UPD	F	Possible ASD
181	PW UPD	M	Possible ASD

Supplementary Table 1. Cell lines used for RNAseq and immunocytochemistry analysis. Asterisk (*) denotes subjects used in RNAseq experiment.

**Gene**	**Function**	**Link to PWS**
PEX10	Peroxisomes breakdown toxins and synthesize lipids	Mutations lead to Zellweger syndrome. Primary phenotypes of Zellweger syndrome are hypotonia and neurodevelopmental delay [80]
SNTB2	Plays a role in regulation of secretory granules and localization of membrane proteins	Dystrophins are associated with muscular dystrophy [81]
CTDP1	Dephosphorylates a subunit of RNApol making it available for transcription	Mutations are associated with neuropathy, facial dysmorphism, and cataracts [82]
AKT1S1	Subunit of mTORC, which regulates cell growth in response to nutrients and hormonal signaling	Altered mTORC signaling has been linked to a variety of neurological disorders including autism, epilepsy, and neurodegenerative disorders [83]

ATP7A	Supplies copper to copper requiring proteins in the secretory pathway	Associated with Menkes disease [84]. Primary phenotypes are hypotonia, hypothermia, failure to thrive, and seizures.
MYL5	Component of ATPase cellular motor protein myosin	Myosin has many neuronal functions, particularly at the synapse [85]
MIPOL1	Function is not well characterized	Link to hand and feet abnormalities
TMEM92	Transmembrane protein with unknown function	Unknown
DHRS1	Functions as an oxidoreductase	Associated with speech/ language impairment

Supplementary Table 2. Core PWS transcripts outside of the 15q11.2-13.1 region that were significantly different versus control in all PWS subgroup.

**Gene**	**Full Name**	**Function**
ACOT9	Acyl-CoA Thioesterase 9	Mitochondrial acyl-CoA thioesterase
AGPAT5	1-Acylglycerol-3-Phosphate O-Acyltransferase 5	Expressed in mitochondria
AIFM1	Apoptosis Inducing Factor Mitochondria Associated 1	NADH oxidoreductase found in the mitochondrial intermembrane space
BNIP1	BCL2 Interacting Protein 1	Involved in mitophagy
CLTC	Clathrin Heavy Chain	Expressed in mitochondria
CMC4	C-X9-C Motif Containing 4	Expressed in mitochondria
COX5B	Cytochrome C Oxidase Subunit 5B	Component of the electron transport chain
COX7B	Cytochrome C Oxidase Subunit 7B	Component of the electron transport chain
COX7C	Cytochrome C Oxidase Subunit 7C	Component of the electron transport chain
CS	Citrate Synthase	TCA cycle enzyme
CYB5A	Cytochrome B5 Type A	Electron carrier expressed in mitochondria

EHHADH	Enoyl-CoA Hydratase And 3-Hydroxyacyl CoA Dehydrogenase	Involved in mitochondrial fatty acid oxidation
FDXR	Ferredoxin Reductase	Mitochondrial flavoprotein involved in the electron transport chain
GFM1	G Elongation Factor Mitochondrial 1	Mitochondrial translation elongation factor
GHR	Growth Hormone Receptor	Expressed in mitochondria
GLUD1	Glutamate Dehydrogenase 1	Mitochondrial matrix enzyme
GPX1	Glutathione Peroxidase 1	Protects cells from oxidative stress
GSTP1	Glutathione S-Transferase Pi 1	Protects cells from oxidative stress
HAP1	Huntingtin Associated Protein 1	Expressed in mitochondria
MFN2	Mitofusin 2	Involved in mitochondrial fusion and maintenance of the mitochondial matrix
MINOS1	Mitochondrial Contact Site And Cristae Organizing System Subunit 10	Involved in the maintenance of mitochondrial architecture
MRPL4	Mitochondrial Ribosomal Protein L4	Component of the mitochondrial ribosomal 39S subunit
MRPL41	Mitochondrial Ribosomal Protein L41	Component of the mitochondrial ribosomal 39S subunit
MRPL47	Mitochondrial Ribosomal Protein L47	Component of the mitochondrial ribosomal 39S subunit
MRPS33	Mitochondrial Ribosomal Protein S33	Component of the mitochondrial ribosomal 28S subunit
MRPS5	Mitochondrial Ribosomal Protein S5	Component of the mitochondrial ribosomal 28S subunit
MTIF2	Mitochondrial Translational Initiation Factor 2	Mitochondrial translation intitation factor
NAXE	NAD(P)HX Epimerase	Involved in mitochondrial metabolic processes

NDUFA1	NADH:Ubiquinone Oxidoreductase Subunit A1	Component of the electron transport chain
NDUFV2	NADH:Ubiquinone Oxidoreductase Core Subunit V2	Component of the electron transport chain
NFS1	Cysteine Desulfurase, Mitochondrial	Expressed in mitochondria
NT5M	5',3'-Nucleotidase, Mitochondrial	5' nucleotidase localized to the mitochondrial matrix
OAT	Ornithine Aminotransferase	Mitochondrial enzyme ornithine aminotransferase
OXLD1	Oxidoreductase Like Domain Containing 1	Expressed in mitochondria
PERP	P53 Apoptosis Effector Related To PMP22	Expressed in mitochondria
POR	Cytochrome P450 Oxidoreductase	Enzyme involved in electron transport
PRKAR2B	Protein Kinase CAMP-Dependent Type II Regulatory Subunit Beta	Plays a role in regulating energy balance
RMND1	Required For Meiotic Nuclear Division 1 Homolog	Involved in mitochondrial translation
SDHC	Succinate Dehydrogenase Complex Subunit C	Component of the electron transport chain
SDHD	Succinate Dehydrogenase Complex Subunit D	Component of the electron transport chain
SLC25A36	Solute Carrier Family 25 Member 36	Mitochondrial transport protein
SLC25A39	Solute Carrier Family 25 Member 39	Mitochondrial transport protein
SLC25A5	Solute Carrier Family 25 Member 5	Mitochondrial transport protein
SLC35F6	Solute Carrier Family 35 Member F6	Involved in the maintenance of mitochondrial membrane potential
SUCLG2	Succinate-CoA Ligase GDP-Forming Subunit Beta	TCA cycle enzyme

SYNE2	Spectrin Repeat Containing Nuclear Envelope Protein 2	Expressed in mitochondria
TIMM17A	Translocase Of Inner Mitochondrial Membrane 17A	Mitochondrial transport protein
TMEM160	Transmembrane Protein 160	Expressed in mitochondria
TMTC1	Transmembrane O-Mannosyltransferase Targeting Cadherins 1	Expressed in mitochondria
TP63	Tumor Protein P63	Expressed in mitochondria
TUFM	Tu Translation Elongation Factor, Mitochondrial	Mitochondrial translation elongation factor
TXN	Thioredoxin	Involved in redox reactions
UQCR10	Ubiquinol-Cytochrome C Reductase, Complex III Subunit X	Component of the electron transport chain
UQCR11	Ubiquinol-Cytochrome C Reductase, Complex III Subunit XI	Component of the electron transport chain
UQCRC2	Ubiquinol-Cytochrome C Reductase Core Protein 2	Component of the electron transport chain
UQCRFS1	Ubiquinol-Cytochrome C Reductase, Rieske Iron-Sulfur Polypeptide 1	Component of the electron transport chain
UQCRH	Ubiquinol-Cytochrome C Reductase Hinge Protein	Component of the electron transport chain
USP30	Ubiquitin Specific Peptidase 30	Involved in mitophagy

Supplementary Table 3. List of genes identified in DAVID mitochondrial enrichment categories.

1.1 Supplementary Figures

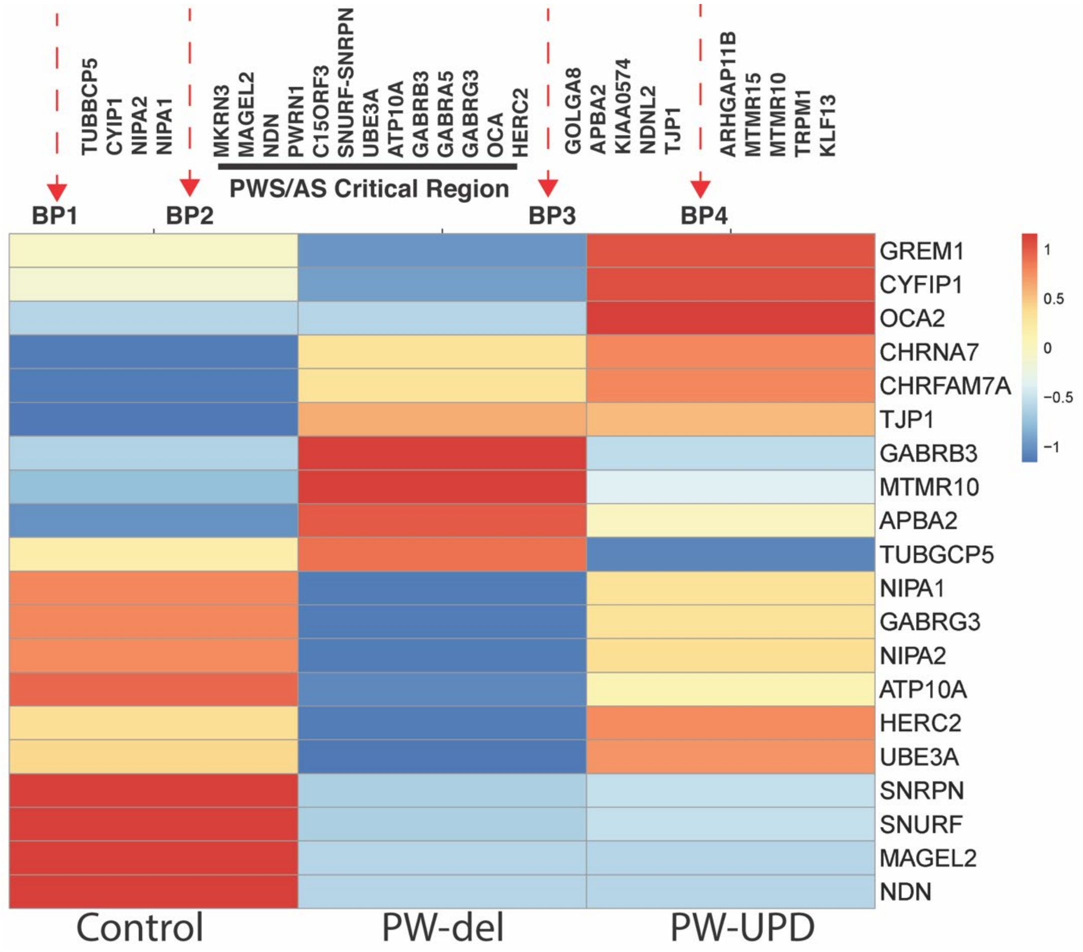

Supplementary Figure 1. Genes in the 15q11.2-q13 critical region shows expected PWS imprinted expression. Across the PWS/AS critical region, maternally imprinted genes such as *MAGEL2*, *SNRPN*, and *SNURF* showed decreased expression in both PW-del and PW-UPD neurons.

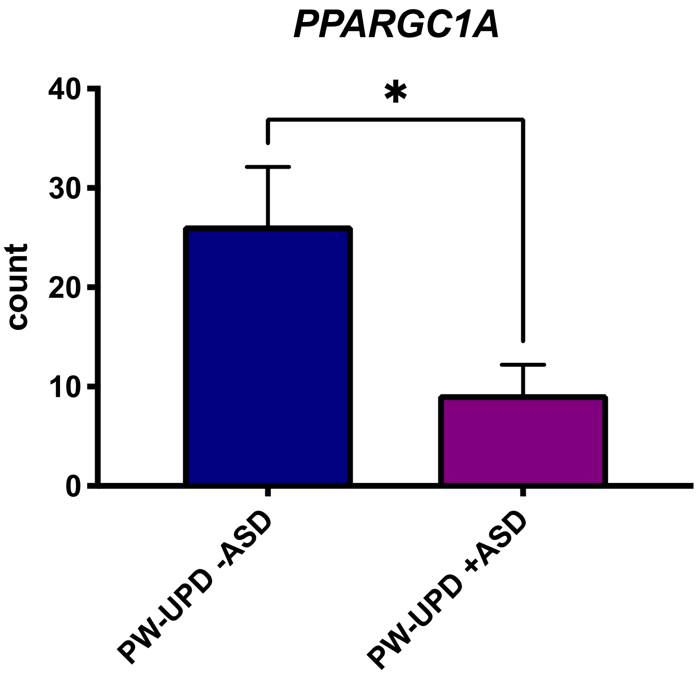

Supplementary Figure 2. Expression of mitochondrial biogenesis factor, *PPARGC1A*, is significantly decreased in PW-UPD +ASD neurons. Mean RNAseq count for each group is shown in the graph. Significance was determined by an unpaired *t*-test ($p \leq 0.05$).
